# Digital PCR to Measure SARS-CoV-2 RNA, Variants, and Outcomes in Youth

**DOI:** 10.1093/jpids/piad101

**Published:** 2023-11-13

**Authors:** Diego R Hijano, Jose A Ferrolino, Zhengming Gu, Jessica N Brazelton, Haiqing Zhu, Sri Suganda, Heather L Glasgow, Ronald H Dallas, Kim J Allison, Gabriela Maron, St. Jude COVID-19 Patients Study Team, Paige Turner, Megan Peterson, Hailey S Ross, Madeline Burton, Sapna Pardasani, Jane S Hankins, Clifford Takemoto, Hiroto Inaba, Sara Helmig, Anna Vinitsky, Melissa R Hines, Ali Y Suliman, Paul G Thomas, E Kaitlynn Allen, Joshua Wolf, Hana Hakim, Nehali Patel, Katherine Knapp, Elisabeth E Adderson, Himani Darji, Li Tang, Thomas P Fabrizio, Richard J Webby, Randall T Hayden

**Affiliations:** Departments of Infectious Diseases, St. Jude Children’s Research Hospital, Memphis, Tennessee, USA; Department of Pediatrics, University of Tennessee Health Science Center, Memphis, Tennessee, USA; Departments of Infectious Diseases, St. Jude Children’s Research Hospital, Memphis, Tennessee, USA; Department of Pathology, St. Jude Children’s Research Hospital, Memphis, Tennessee, USA; Department of Pathology, St. Jude Children’s Research Hospital, Memphis, Tennessee, USA; Department of Pathology, St. Jude Children’s Research Hospital, Memphis, Tennessee, USA; Department of Pathology, St. Jude Children’s Research Hospital, Memphis, Tennessee, USA; Department of Pathology, St. Jude Children’s Research Hospital, Memphis, Tennessee, USA; Departments of Infectious Diseases, St. Jude Children’s Research Hospital, Memphis, Tennessee, USA; Departments of Infectious Diseases, St. Jude Children’s Research Hospital, Memphis, Tennessee, USA; Departments of Infectious Diseases, St. Jude Children’s Research Hospital, Memphis, Tennessee, USA; Department of Pediatrics, University of Tennessee Health Science Center, Memphis, Tennessee, USA; Departments of Infectious Diseases, St. Jude Children’s Research Hospital, Memphis, TN, USA; Departments of Infectious Diseases, St. Jude Children’s Research Hospital, Memphis, TN, USA; Departments of Infectious Diseases, St. Jude Children’s Research Hospital, Memphis, TN, USA; Departments of Infectious Diseases, St. Jude Children’s Research Hospital, Memphis, TN, USA; Departments of Infectious Diseases, St. Jude Children’s Research Hospital, Memphis, TN, USA; Departments of Hematology, St. Jude Children’s Research Hospital, Memphis, TN, USA; Departments of Global Pediatric Medicine, St. Jude Children’s Research Hospital, Memphis, TN, USA; Departments of Hematology, St. Jude Children’s Research Hospital, Memphis, TN, USA; Departments of Oncology, St. Jude Children’s Research Hospital, Memphis, TN, USA; Departments of Oncology, St. Jude Children’s Research Hospital, Memphis, TN, USA; Departments of Oncology, St. Jude Children’s Research Hospital, Memphis, TN, USA; Departments of Pediatric Medicine, St. Jude Children’s Research Hospital, Memphis, TN, USA; Departments of Bone Marrow Transplant & Cell Therapy, St. Jude Children’s Research Hospital, Memphis, TN, USA; Departments of Immunology St. Jude Children’s Research Hospital, Memphis, TN, USA; Departments of Immunology St. Jude Children’s Research Hospital, Memphis, TN, USA; Departments of Infectious Diseases, St. Jude Children’s Research Hospital, Memphis, TN, USA; Departments of Infectious Diseases, St. Jude Children’s Research Hospital, Memphis, TN, USA; Department of Preventive Medicine, University of Tennessee Health Science Center, Memphis, TN, USA; Departments of Infectious Diseases, St. Jude Children’s Research Hospital, Memphis, TN, USA; Departments of Infectious Diseases, St. Jude Children’s Research Hospital, Memphis, TN, USA; Departments of Infectious Diseases, St. Jude Children’s Research Hospital, Memphis, TN, USA; Department of Pediatrics, University of Tennessee Health Science Center, Memphis, TN, USA; Department of Biostatistics, St. Jude Children’s Research Hospital, Memphis, Tennessee, USA; Department of Biostatistics, St. Jude Children’s Research Hospital, Memphis, Tennessee, USA; Departments of Infectious Diseases, St. Jude Children’s Research Hospital, Memphis, Tennessee, USA; Departments of Infectious Diseases, St. Jude Children’s Research Hospital, Memphis, Tennessee, USA; Department of Pathology, St. Jude Children’s Research Hospital, Memphis, Tennessee, USA

**Keywords:** cancer, children, COVID-19, Ct values, digital PCR, International Units, SARS-CoV-2, sickle cell disease, viral load

## Abstract

**Background:**

The role of SARS-CoV-2 viral load in predicting contagiousness, disease severity, transmissibility, and clinical decision-making continues to be an area of great interest. However, most studies have been in adults and have evaluated SARS-CoV-2 loads using cycle thresholds (Ct) values, which are not standardized preventing consistent interpretation critical to understanding clinical impact and utility. Here, a quantitative SARS-CoV-2 reverse-transcription digital PCR (RT-dPCR) assay normalized to WHO International Units was applied to children at risk of severe disease diagnosed with COVID-19 at St. Jude Children’s Research Hospital between March 28, 2020, and January 31, 2022.

**Methods:**

Demographic and clinical information from children, adolescents, and young adults treated at St. Jude Children’s Research Hospital were abstracted from medical records. Respiratory samples underwent SARS-CoV-2 RNA quantitation by RT-dPCR targeting N1 and N2 genes, with sequencing to determine the genetic lineage of infecting virus.

**Results:**

Four hundred and sixty-two patients aged 0–24 years (median 11 years old) were included during the study period. Most patients were infected by the omicron variant (43.72%), followed by ancestral strain (22.29%), delta (13.20%), and alpha (2.16%). Viral load at presentation ranged from 2.49 to 9.14 log_10_ IU/mL, and higher viral RNA loads were associated with symptoms (OR 1.32; CI 95% 1.16–1.49) and respiratory disease (OR 1.23; CI 95% 1.07–1.41). Viral load did not differ by SARS-CoV-2 variant, vaccination status, age, or baseline diagnosis.

**Conclusions:**

SARS-CoV-2 RNA loads predict the presence of symptomatic and respiratory diseases. The use of standardized, quantitative methods is feasible, allows for replication, and comparisons across institutions, and has the potential to facilitate consensus quantitative thresholds for risk stratification and treatment.

## INTRODUCTION

As of May 2023, more than 15 million children have been infected with SARS-CoV-2, with over 8000 new cases per week in the United States [1]. While repeated exposures, vaccines, and therapeutic options have significantly affected the course of the COVID-19 pandemic, rapid viral evolution, vaccine hesitancy, and vaccine inequity have contributed to SARS-CoV-2 remaining one of the most common causes of childhood mortality in the United States [2, 3].

The role of SARS-CoV-2 viral load in disease severity and transmission, and correlation with clinical disease remain unknown, partly due to the widespread use of cycle threshold (Ct) values as indicators of viral load in research and clinical practice [4–6]. Although there is a relative relationship between Ct values and the amount of virus RNA in a clinical specimen, Ct values generated by qualitative PCR tests are not considered reliable measures of viral load [7–9]. The use of RT-PCR Ct values as a surrogate of RNA concentration may introduce inaccuracy because these values may not have a linear correlation with the quantity of viral RNA across the analytical measurement range (AMR) of a given assay, a critical characteristic when measuring samples with either very high or very low RNA loads [7]. Within-assay variability across the AMR may also be significant. Ct values are not normalized to known nucleic acid concentrations, and qualitative assays reporting Ct values have poor inter-assay agreement and lack linear correlation, limiting the generalization of results [8]. Despite caution advised by the Infectious Diseases Society of America, the Association of Molecular Pathology, and other scientific societies against the presentation of clinical data using Ct values or use Ct values to predict active infection, disease severity, or transmissibility, Ct values continue to be reported and used [10]. Consequently, while studies have assessed the role of viral load in COVID-19 in children, these data are difficult to replicate, apply uniformly, compare, or generalize [11–16].

Digital PCR (dPCR) provides reproducible, highly accurate results without the need for quantitative calibrators [17, 18]. Furthermore, the use of international quantitative standards has helped to harmonize interlaboratory viral nucleic acid load determinations in other settings, including their use for transplant-related viruses such as CMV and EBV [19, 20]. Here we describe the use of a quantitative SARS-CoV-2 digital reverse-transcription PCR (RT-dPCR) assay normalized to international units in children at high risk of severe respiratory disease over the course of the COVID-19 pandemic. The association of age, baseline diagnosis, vaccination status, and genetic variants on viral load and correlation it with clinical presentation and outcomes are assessed using a standardized assay with the goal of facilitating consistent results and meaningful clinical application.

## METHODS

### Patients and Clinical Information

All patients diagnosed with SARS-CoV-2 infection in outpatient and inpatient settings at St. Jude between March 28, 2020 and January 31, 2022, were included for the study. Demographics, medical history, clinical presentation, and infection outcomes were abstracted from the electronic medical record. Remnant aliquots of nasal or nasopharyngeal samples that had tested positive for SARS-CoV-2 by a qualitative assay for routine clinical diagnosis were frozen at −70°C and thawed at room temperature prior to quantification and sequencing. Testing was performed using 1 of 3 test systems: the NeuMoDx™ SARS-CoV-2 Assay, (Qiagen, Hilden, Germany), the Roche Cobas6800/8800 assay (Roche Diagnostics, Risch-Rotkreuz, Switzerland), or the altona RealStar® SARS-COV-2 RT-PCR assay (altona Diagnostics, Hamburg, Germany), each of which had received emergency use authorization (EUA) by the US Food and Drug Administration (US FDA). All 3 methods had undergone validation by the St. Jude Clinical COVID Laboratory and been shown to perform as expected, with comparable accuracy across all systems. The clinical sample was collected in universal transport medium. A total of 50 µL of nucleic acid was eluted from 200 µL of extracted clinical specimen using the MagMax™ 96 AI/ND Viral RNA Isolation Kit (ThermoFisher) and KingFisher Flex instrument (ThermoFisher) according to the manufacturer’s recommendation. The study was reviewed and approved by The St. Jude Institutional Review Board.

### SARS-CoV-2 RT-dPCR Assay

The Bio-Rad SARS-CoV-2 RT-dPCR Test (Bio-Rad Laboratories, Inc., CA, USA), which was authorized for emergency use by the Food and Drug Administration (FDA) was used for quantitative detection of SARS-CoV-2 RNA. Viral RNA loads were generated for both N1 and N2 targets. As both targets showed nearly identical results, data from the N1 target are reported. Results were normalized to the 20/146 First WHO International Standard for SARS-CoV-2 RNA (product code 20/146, National Institute for Biological Standards and Control, South Mimms, Potters Bar, United Kingdom), as previously reported, to produce data in log_10_ IU/mL [21]. Lower limits of quantification (LLoQ) and limits of detection for the assay were the same: 3.84 log_10_ IU/mL for N1 and 3.82 log_10_ IU/mL for N2 with an upper limit of quantification of 7.86 log_10_ IU/mL and 7.88 log_10_ IU/mL for N1 and N2, respectively [21].

### SARS-CoV-2 Sequencing Assay

Paired-end sequencing was performed on a MiSeq II or NextSeq 500 (Illumina, Inc., San Diego, CA), using Swift Normalase® Amplicon Panel (SNAP) SARS-CoV-2 Additional Genome Coverage, and SARS-CoV-2 S Gene Panels (Swift Biosciences, Ann Arbor, MI) as previously described [22]. Sequencing was performed on initial sample and every 30 days for patients who continue to test positive over time. Analysis was performed using an internally developed computational pipeline (idCOV, Center for Applied Bioinformatics, St. Jude) [23] and a commercial pipeline (COSMOSID, Rockville, MD), and variant determination was performed by consensus between the 2 pipelines.

### Statistical Analysis

Patient characteristics were summarized by descriptive statistics at each time interval. Frequency and proportions were calculated for categorical variables. Means and standard deviations (or medians and interquartile ranges) were calculated for continuous variables. Collection time of first and follow-up samples was divided into 4-time intervals: days 0 (baseline)–6, days 7–13, days 14–27, and ≥28 days. SARS-CoV-2 load, and absolute lymphocyte count (ALC) were reported using a log_10_ scale. Simple linear regression was used to compute values of positive controls reported in copies/mL against corresponding nominal values in IU/mL and conversion of viral RNA load from copies/mL into IU/mL units was performed as previously reported (5 copies/mL was equivalent to 1 IU/mL) [21]. Samples that were positive below the LLoQ were assigned a viral load midway between 0 and the LLoQ for the purposes of analysis. A multivariate stepwise logistic regression model was run and adjusted for the following variables known to affect disease severity: age, gender, race, cancer, chemotherapy, sequencing variant, ALC, and vaccination status [24–29]. The adjusted variables with *P* value <.1 were selected in the model. Receiver operating characteristics (ROC) curves were performed to determine the value of viral RNA load in predicting symptoms and clinical outcomes. Optimal cut-point levels were derived using the highest Youden indices (J). All analyses were performed using SAS 9.4 (SAS Institute Inc. 2013. SAS® 9.4 Statements: Reference. Cary, NC: SAS Institute Inc.) and R 4.2.0 (R Core Team (2020). R: A language and environment for statistical computing. R Foundation for Statistical Computing, Vienna, Austria. https://www.R-project.org/).

## RESULTS

### Demographics

During the study period, 462 patients tested positive for SARS-CoV-2 and contributed 628 samples. Day 0 was defined as the day of symptom onset or day of first positive SARS-CoV-2 test if the individual was asymptomatic. Among positive samples, 435 samples were collected during the first week of infection (days 0–6), 76 samples (from 67 patients) were collected between days 7–13, and 86 samples (from 66 patients), 14–27 days from symptom onset or diagnosis. Only 31 samples (from 22 patients) were obtained ≥28 days from infection. A majority of samples (*n* = 342; 74%) were collected by mid-turbinate swab, with the rest by nasopharyngeal swab. Median patient age was 11 years (IQR [5–15]); 45.02% were female and most were non-Hispanic (86.58%) and Black (52.81%) (Table 1). Over half of patients (53.03%) had a diagnosis of cancer, of whom 40.82% were receiving chemotherapy at the time of infection. A total of 140 patients (30.30%) had sickle cell disease (SCD), and 26 (5.63%) had HIV. All patients with HIV were receiving antiretroviral therapy; most (65.38%) had undetectable HIV loads, and all had CD4 count above 200/mm^3^. Only 93 (20.13%) patients had received 1 or more doses of SARS-CoV-2 vaccine prior to infection, including Pfizer-BioNTech (*n* = 67), Janssen (*n* = 18), and Moderna (*n* = 10). Of these, 13 (13.98%) received 3 or more vaccine doses prior to infection.

**Table 1.** Demographic and Clinical Characteristic of Individuals With SARS-CoV-2 Infection

Total Patients	462
Age (median [range]) in years	11 [0–24]
Sex (%)	
Female	208 (45.02)
Male	254 (54.98)
Race (%)	
Black	244 (52.81)
White	187 (40.48)
Multiracial	20 (4.33)
Asian	5 (1.08)
Unknown	1 (0.22)
Other	5 (1.08)
Ethnicity (%)	
Non-Hispanic/non-Latino	400 (86.58)
Hispanic/Latino	50 (10.82)
Not specified	12 (2.60)
Diagnosis (%)	
HIV	26 (5.63)
Leukemia/Lymphoma	87 (18.83)
SCD	140 (30.30)
ST/NO	145 (31.39)
Other^a^	64 (13.85)
Cancer diagnosis (%)	
Yes	245 (53.03)
No	217 (46.97)
Received chemotherapy (%)	
Yes	100 (40.82)
No	145 (59.18)
SARS-COV-2 vaccination prior to infection (%)	
No	369 (79.87)
Yes	93 (20.13)
Number of vaccine doses (median [IQR])	2.00 [1.00, 2.00]
Pfizer-BioNTech (%)	
Yes	67 (72.04)
Moderna (%)	
Yes	10 (10.75)
Janssen (%)	
Yes	18 (19.35)
Reason for testing (%)	
Symptoms	172 (37.23)
Asymptomatic screening	290 (62.77)
Did the patient become symptomatic? (%)	
No	205 (70.69)
Yes	85 (29.31)
Was episode initial or a reinfection (%)	
Initial infection	454 (98.27)
Reinfection	8 (1.73)
Days from previous infectious episode (median [IQR])	91.00 [74.25, 142.50]
SARS-CoV2- Variant (%)	
Omicron	202 (43.72)
Ancestral	103 (22.29)
Delta	61 (13.20)
Alpha	10 (2.16)
Epsilon	1 (0.22)
Undetermined	85 (18.40)
Symptoms within 28 days of Day 0? (%)	
Yes	247 (53.46)
No	215 (46.54)
Fever (%)	
Yes	155 (62.75)
No	92 (37.25)
Headache (%)	
Yes	47 (19.03)
No	200 (80.97)
Cough (%)	
Yes	157 (63.56)
No	90 (36.44)
Sore throat (%)	
Yes	42 (17.00)
No	205 (83.00)
Loss of smell or taste (%)	
Yes	26 (10.53)
No	221 (89.47)
Shortness of breath (%)	
Yes	22 (8.91)
No	225 (91.09)
Diarrhea (%)	
Yes	20 (8.10)
No	227 (91.90)
Hospitalization (%)	
Yes	51 (11.04)
Already hospitalized for reasons other than COVID-19	26 (5.63)
No	385 (83.33)
ICU (%)	
Yes	8 (1.73)
No	69 (14.94)
Not hospitalized	385 (83.33)
Any respiratory symptoms at Day 0? (%)	
No	281 (61.22)
Yes	178 (38.78)
Respiratory symptoms at Day 0 (%)	
LRTI	17 (9.55)
URTI	161 (90.45)
Progression to LRTI (%)	
No	440 (99.55)
Unknown	1 (0.23)
Yes	1 (0.23)
Death (%)	
Yes	2 (0.43)
No	460 (99.57)
MIS-C (%)	
Yes	1 (0.26)
No	380 (99.74)
Received steroids within 28 days (%)	
Yes	12 (2.65)
No	440 (97.35)
Received convalescent plasma within 28 days (%)	
Yes	2 (0.43)
No	460 (99.57)
Received remdesivir within 28 days (%)	
Yes	24 (5.19)
No	438 (94.81)
Remdesivir usage (%)	
Prevent progression	15 (62.50)
Treatment of severe COVID-19	9 (37.50)
Duration of remdesivir use (median [IQR])	3.00 [3.00, 5.00]

^a^For a complete list of conditions included in Other please refer to Supplementary Table 16.

### Clinical Presentation and Outcome

A total of 290 (62.87%) patients were asymptomatic at the time of testing, and 172 (37.23) were tested due to symptoms concerning COVID-19 (Table 1). Among those who were asymptomatic, 85 (29.30%) subsequently became symptomatic. Fever and cough were most commonly reported, followed by headache and sore throat. (Table 1). Lost sense of taste or smell, diarrhea, and shortness of breath were reported in 10.53%, 8.10%, and 8.91%, respectively. Fifty-one (11.04%) patients were hospitalized due to COVID-19, 8 (1.73%) required ICU admission, and 2 (0.43%) patients died due to progressive cancer with no relation to SARS-CoV-2 infection. Twenty-one (16.80%) patients with SCD presented with acute chest syndrome. Omicron was the most frequently identified variant (43.72% of samples) followed by Ancestral strain (22.29%) and delta (13.20%). Alpha variant was only found in 2.16% of samples. In 18.40% of samples the responsible variant could not be determined due to poor sequence quality (Supplementary Table 1). Only 1 patient met CDC criteria for multisystemic inflammatory syndrome (MIS).

**Figure 1.**
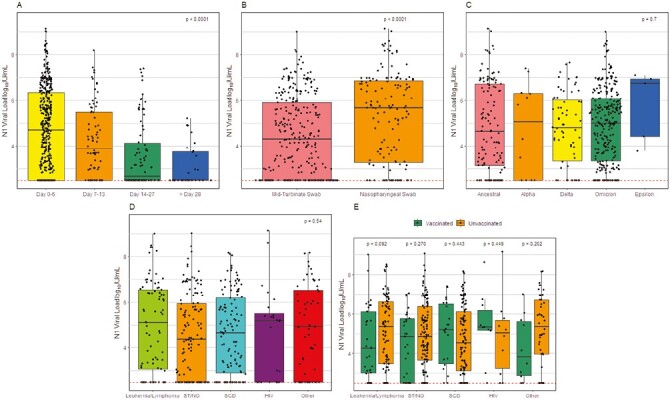
SARS-CoV-2 viral load (N1 log_10_ IU/mL) in children. A, SARS-CoV-2 loads over time. B, SARS-CoV-2 loads by sample type. C, SARS-CoV-2 loads by variant of concern. D, SARS-CoV-2 loads by underlying disorder. E, SARS-CoV-2 loads by vaccination status prior to infection and underlying disorder. Dotted line represents the demonstrated lower limits of the assay analytical measurement range.

The nature of clinical presentation and infection outcomes did not differ significantly among patients with and without cancer (Supplementary Table 2) although patients without malignancy were more likely to have fever and were more frequently hospitalized due to COVID-19. Although not significant, patients with SCD developed lower respiratory tract infections (LRTI) and required hospitalization due to COVID-19 more frequently than those with cancer. The need for ICU and steroids those with SCD was similar to those with hematological malignancies, and higher than in patients with solid or brain tumors. While patients with SCD had significantly higher lymphocyte count, no difference was observed in SARS-CoV-2 loads (Table 2). In contrast, patients with cancer were more likely to be already hospitalized for other reasons, such as planned chemotherapy, when diagnosed with COVID-19. When assessing differences in illness across SARS-CoV-2 variants, the proportion of patients with fever was not significantly different (*P* = .13). Diarrhea was less frequently reported in patients with the omicron variant in comparison with those with delta and ancestral variants (*P* = .076). LRTI were rare, but most frequently seen in patients with the delta variant. No differences were seen in proportions of hospitalization, the need for ICU care, or in mortality (Supplementary Table 3). The median SARS-CoV-2 load upon presentation of all patients was 4.66 log_10_ IU/mL (IQR: 2.49–6.36) and decreased over time after admission (Figure 1A). Samples collected by nasopharyngeal swab had significantly higher SARS-CoV-2 loads (median 5.66; IQR 3.36, 6.86) than those collected by mid-turbinate swab (median 4.30; IQR 2.49, 5.90); *P* < .001 (Figure 1B). Viral loads of initially collected samples did not differ by viral genetic lineage (Figure 1C), underlying disease (Figure 1D), vaccination status (Figure 1E), type of vaccine, or number of doses of vaccine prior to infection (Supplementary Table 4).

**Table 2.** Comparison of Symptoms, Clinical Outcomes, and Laboratory Values of Patients With SARS-CoV-2 With SCD and Malignancy

		Malignancy	Sickle Cell Disease	P
Variable	Categories	(*n* = 232)	(*n* = 140)	
Fever	No	171 (73.71)	63 (45.00)	<.0001
	Yes	61 (26.29)	77 (55.00)	
Headache	No	212 (91.38)	118 (84.29)	.0542
	Yes	20 (8.62)	22 (15.71)	
Cough	No	163 (70.26)	75 (53.57)	.0017
	Yes	69 (29.74)	65 (46.43)	
Sore throat	No	215 (92.67)	122 (87.14)	.1126
	Yes	17 (7.33)	18 (12.86)	
Loss of taste and smell	No	218 (93.97)	132 (94.29)	1
	Yes	14 (6.03)	8 (5.71)	
Shortness of breath	No	224 (96.55)	129 (92.14)	.1035
	Yes	8 (3.45)	11 (7.86)	
Diarrhea	No	219 (94.40)	136 (97.14)	.3308
	Yes	13 (5.60)	4 (2.86)	
LRTI/URTI	LRTI	4 (1.72)	11 (7.86)	.0137
	None	140 (60.34)	77 (55.00)	
	URTI	88 (37.93)	52 (37.14)	
Hospitalization	Already hospitalized	24 (10.34)	2 (1.43)	<.0001
	No	194 (83.62)	105 (75.00)	
	Yes	14 (6.03)	33 (23.57)	
ICU	No	35 (15.09)	31 (22.14)	.1112
	Not hospitalized	194 (83.62)	105 (75.00)	
	Yes	3 (1.29)	4 (2.86)	
Death	No	230 (99.14)	140 (100.00)	.7115
	Yes	2 (0.86)	0 (0.00)	
Treatment convalescent Plasma	No	230 (99.14)	140 (100.00)	.7115
	Yes	2 (0.86)	0 (0.00)	
Treatment remdesivir	No	222 (95.69)	128 (91.43)	.144
	Yes	10 (4.31)	12 (8.57)	
Treatment steroid	No	222 (98.23)	131 (94.93)	.1415
	Yes	4 (1.77)	7 (5.07)	
Vaccination	No	186 (80.17)	116 (82.86)	.6136
	Yes	46 (19.83)	24 (17.14)	
Sequencing	Alpha	9 (3.88)	3 (2.14)	.6274
	Ancestral	53 (22.84)	35 (25.00)	
	Delta	32 (13.79)	13 (9.29)	
	Epsilon	1 (0.43)	0 (0.00)	
	Omicron	94 (40.52)	63 (45.00)	
	Undetermined	43 (18.53)	26 (18.57)	
ALC	Mean (SD)	1711.14 (1632.60)	3234.00 (2724.69)	<.0001
	Median (IQR)	1268.50 [519.75, 2382.50]	2762.50 [1705.25, 3693.50]	<.0001
Log_10_ IU/mL SARS-CoV-2 N1	Mean (SD)	4.59 (1.87)	4.41 (1.77)	.3548
	Median (IQR)	4.37 [2.49, 6.34]	4.06 [2.49, 5.84]	.4803

Twenty-seven patients had 2 episodes of COVID-19. The median time between episodes was 193 days (range 30–577 days). Most patients’ initial episode was caused by ancestral strain (40.74%) and reinfection by omicron (70.37%). Five patients had an initial episode caused by delta and reinfection with omicron (Supplementary Table 5). One patient was originally infected with delta (viral load 6.68 log_10_ IU/mL). After 32 days and several negative SARS-CoV-2 PCR tests, he tested positive again with same variant (viral load 6.95 log_10_ IU/mL). It was unclear whether this represented reinfection or relapse, given that there was a month interval between positive samples, and several intervening negative tests. His viral load subsequently declined (2.95 log_10_ IU/mL) and became negative over the following 14 days. Over 90 days after the last positive SARS-CoV-2 test, he tested positive again for SARS-CoV-2, omicron variant (initial viral load 4.72 log_10_ IU/mL).

Twenty-four patients (5.19%) received remdesivir; 15 (62.50%) to prevent progression to LRTI, and 9 (37.50%) for treatment of severe COVID-19. The median duration of remdesivir was 3 days (3–5 days). No other antivirals were prescribed. Twelve patients (2.65%) received steroids, and 2 patients (0.43%) received convalescent plasma.

### SARS-CoV-2 Load, Symptoms, and Outcomes

Viral RNA load in initial positive samples was associated with symptom development in individuals who were asymptomatic at the time of diagnosis irrespective of age, gender, and race (OR 1.18; 95% CI 1.02–1.37; *P* value .026) (Supplementary Tables 6 and 7). ROC analysis showed that a viral RNA load of 4.86 log_10_ IU/mL or greater upon initially asymptomatic presentation was associated with subsequent development of symptoms with a sensitivity of 36.47% and a specificity of 81.46% (AUC: 0.58). Using maximum viral load during the course of infection, sensitivity was improved up to 64.71% specificity decreased to 58.05% (Supplementary Table 8, Supplementary Figure 2).

Higher viral RNA load at presentation was significantly associated with symptomatic disease (OR 1.34; CI 95% 1.20–1.50; *P* <.0001), and respiratory tract infection symptoms (OR 1.25; CI 95% 1.11–1.39; *P* value <.0001). These associations were seen irrespective of age, race, presence of malignancy, receipt of chemotherapy, ALC, receipt of vaccination, or differences in infecting variants (Supplementary Table 9; Table 3). Median viral RNA load at presentation, in symptomatic individuals (4.56 log_10_ IU/mL; IQR: 2.49–6.43) was higher compared with those without symptoms (3.65 log_10_ IU/mL; IQR: 2.49–5.54), *P* = .002. However, viral RNA load at symptom onset did not significantly correlate with hospitalization or severe disease (Supplementary Table 10). Similar results were observed for maximum viral load at any timepoint (Supplementary Tables 11 and 12) and in linear mixed effect models accounting for multiple samples (Supplementary Tables 13–16).

**Table 3.** Odds Ratio of Various Clinical Outcomes Based on Initial SARS-CoV-2 Loads

	Odds ratio (95% CI)	*P* value	Odds ratio (95% CI) adjusted for age	*P* value	Odds ratio (95% CI) ­adjusted for race^a^	*P* value	Odds ratio (95% CI) adjusted for cancer	*P* value	Odds ratio (95% CI) adjusted for chemotherapy	*P* value	Odds ratio (95% CI) adjusted for Lymphocyte counts (log value)	*P* value	Odds ratio (95% CI) adjusted for sequencing variant^b^	*P* value	Odds ratio (95% CI) adjusted for vaccine status	*P* value
SARS-CoV-2 N1																
URTI/LRTI																
RTI vs None	1.25 (1.11–1.39)	.0001	1.25 (1.12–1.40)	.0001	1.25 (1.12–1.40)	.0001	1.24 (1.11–1.39)	.0001	1.24 (1.11–1.39)	.0002	1.25 (1.11–1.41)	.0003	1.23 (1.09–1.40)	.0012	1.24 (1.11–1.39)	.0001
Hospitalization																
Yes vs No	1.13 (0.94–1.35)	.1877	1.13 (0.95–1.35)	.1745	1.13 (0.94–1.35)	.2006	1.13 (0.95–1.36)	.1741	1.13 (0.94–1.35)	.1868	1.11 (0.93–1.33)	.2460	1.07 (0.88–1.30)	.5051	1.13 (0.95–1.35)	.1803
ICU admission																
Not admitted to ICU vs Not hospitalized	1.08 (0.92–1.26)	.3589	1.06 (0.91–1.25)	.4590	1.08 (0.92–1.26)	.3536	1.08 (0.92–1.26)	.3641	1.04 (0.89–1.22)	.6184	1.04 (0.88–1.22)	.6352	1.02 (0.86–1.21)	.8309	1.08 (0.92–1.26)	.3567
Yes vs Not hospitalized	1.46 (0.95–2.24)	.0814	1.46 (0.96–2.24)	.0797	1.49 (0.96–2.30)	.0733	1.47 (0.96–2.26)	.0790	1.48 (0.97–2.27)	.0711	1.45 (0.94–2.22)	.0892	1.29 (0.83–2.00)	.2576	1.47 (0.96–2.26)	.0790

Abbreviation: RTI, respiratory tract infection.

^a^Race here is White and Asian vs Black.

^b^Sequencing variant is grouped as Omicron, Ancestral, Delta, and Others (Alpha, Epsilon and Undetermined).

## DISCUSSION

We describe implementation of a standardized quantitative SARS-CoV-2 RT-dPCR assay, normalized to the first WHO International Standard for SARS-CoV-2 RNA, and correlation of assay results with symptomatic disease in a large cohort of children and young adults, most of whom had an underlying medical condition placing them at risk for development of severe disease.

As previously reported in adults, and in agreement with other groups, higher viral load was associated with increased likelihood of symptoms and respiratory disease. In contrast to our previous observations in adults, viral load poorly predicted which asymptomatic patients would subsequently develop symptoms [11–13, 21, 30]. Additional work in this area could help determine optimal thresholds to initiate treatment in these populations.

Reports of the ability of viral load to predict severe disease are contradictory [15, 31]. In our cohort of children at risk for severe COVID-19, we found no difference in viral loads among those requiring hospitalization compared with those managed as outpatients. Further research using standardized, quantitative assays, as reported here, will help better inform the role of viral load in predicting severe disease among other patient groups.

Initial reports examining the association of viral variants and viral load found that the delta variant established infection and spread faster as a result of higher viral loads in the respiratory tract [32, 33]. Subsequent studies, however, did not confirm these differences [34]. Consistent with the latter, we observed no significant difference in viral RNA loads across variants. A possible explanation is the variability in timing of the first sample in relation to symptom onset, as viral load peaks with development of symptoms onset and subsequently decreases over the course of illness [35]. Likewise, studies assessing the impact of age, race, and prior COVID-19 vaccination on viral load have yielded conflicting results [27, 28, 36–39]. In this cohort, these variables did not influence viral RNA loads.

At the time of this publication, no SARS-CoV-2 quantitative test is cleared or approved for clinical diagnostic use by the FDA. While intuitive to many clinicians, the use of Ct values, as surrogate measures of RNA viral load, should continue to be strongly discouraged due to their poor precision and lack of standardization. One study showed that FDA EUA qualitative test methods reporting Ct values of identical control materials varied by as much as 14 cycles [10]. The reproducibility of assays tested on the same instrument differed by a median of 3 cycles [8, 10]. Reporting viral quantity is clearly more useful than Ct values, especially when values are normalized to a common quantitative standard, facilitating interpretation, reproducibility, and subsequent establishment of consensus treatment thresholds that so far remain elusive. However, the time and resources required to validate quantitative assays can be a limiting factor to wide adoption of these tests. Quantitation of initial samples in patients at high risk for severe COVID-19, as well as follow-up samples in those receiving antivirals may be a judicious first step in using this test for clinical care. However, additional studies are needed to make specific clinical recommendations regarding frequency of testing.

This study has several limitations. First, the timing of samples in reference to onset of disease varied from patient to patient by a few days during each interval collected. Day-to-day variation in RNA load and declining loads over time as individuals recover may account for some of our findings. To ensure the accuracy of the results, a variety of clinical samples were tested during the assay validation process, which aimed to demonstrate consistent performance across patients. The testing process included an internal positive control whose detection at a specific level confirmed the absence of assay inhibition. Sample aliquots were frozen and only thawed once prior to measurement of viral load to minimize freeze/thaw cycles that can affect viral loads. SARS-CoV-2 RT-dPCR Ct values have demonstrated stability with repetitive freeze-thawing and prolonged storage, suggesting viral load measurements performed here were unlikely to be affected [40]. All staff involved in sample collection underwent proper training in collection techniques and competency assessment. Despite these measures and the use of a standardized protocol for sample collection, some sampling variability is expected and may have affected assay results. Quantitation of RNA viruses cannot discern between virus and viral transcripts, potentially introducing a further limitation to result interpretation. There were insufficient numbers of patients with severe disease to adequately assess the correlation between viral load and severity of infection. Finally, this was a single-center study; clinical information was abstracted retrospectively from the electronic medical records and, therefore, subject to recording bias.

Strengths of this study include the large size of our cohort and inclusion of children with underlying diseases that may increase risk for severe COVID-19. Samples were obtained from all major waves of the pandemic. We used a robustly validated RT-dPCR assay with stringently applied requirements for internal positive control detection at a predefined level to demonstrate lack of assay inhibition. In addition, training and competency assessment for sample collection was performed for all staff to minimize variability during sample collection and processing. Finally, commercially available reagents were utilized on a widely available RT-dPCR platform, allowing for implementation of this method in any laboratory running high-complexity molecular testing for clinical purposes.

Using standardized RT-dPCR methods to improve accuracy and reproducibility and to facilitate quantitative equivalency across different testing locations represents a step forward in the field of SARS-CoV-2 RNA load measurement. This work lays the foundation for additional studies by providing new insights on the implications of SARS-CoV-2 viral RNA load in vulnerable pediatric patients.

St. Jude COVID-19 Patients Study Team members

Paige Turner^1^, Megan Peterson^1^, Hailey S. Ross^1^, Madeline Burton^1^, Sapna Pardasani^1^, Jane S Hankins^2,3^, Clifford Takemoto^2^, Hiroto Inaba^4^, Sara Helmig^4^, Anna Vinitsky^4^, Melissa R Hines^5^, Ali Y Suliman^6^, Paul G Thomas^7^, E Kaitlynn Allen^7^, Joshua Wolf^1^, Hana Hakim^1,8^, Nehali Patel^1^, Katherine Knapp^1^ and Elisabeth E. Adderson^1,9^.

^1^Departments of Infectious Diseases, ^2^Hematology, ^3^Global Pediatric Medicine, ^4^Oncology, ^5^Pediatric Medicine, ^6^Bone Marrow Transplant & Cell Therapy, ^7^Immunology St. Jude Children’s Research Hospital, Memphis, TN, USA. ^8^Department of Preventive Medicine, University of Tennessee Health Science Center, Memphis, TN, USA. ^9^Department of Pediatrics, University of Tennessee Health Science Center, Memphis, TN, USA.

## Supplementary Material

piad101_suppl_Supplementary_Figures

piad101_suppl_Supplementary_Tables

## Data Availability

De-identified individual participant data will not be made available.

## Notes

***Financial support*.** This study was supported by the American Lebanese Syrian Associated Charities and St. Jude Children’s Research Hospital. Bio-Rad Laboratories, Inc. provided PCR reagents for this study. No additional external funding was used in design or conduct of the study; the collection, management, analysis, or interpretation of the data; the preparation, review, or approval of the manuscript; or decision to submit the manuscript for publication.

***Potential conflicts of interest*.** R. T. H. has served on advisory boards for Abbott Diagnostics and T2 Diagnostics. All other authors report no potential conflicts.
