# Supplementary material for: Digital PCR to Measure SARS-CoV-2 RNA, Variants, and Outcomes in Youth
Source: J Pediatric Infect Dis Soc. 2023 Nov 13;12(12):618–26. doi: 10.1093/jpids/piad101 (PMC10725239; doi:10.1093/jpids/piad101)

Supplementary Figure 1: Area under the curve (AUC) for asymptomatic patients who became symptomatic versus patients who remained asymptomatic throughout infection.

Supplementary Figure 2: SARS-CoV-2 loads and risks for severe COVID-19. Odds ratio (OR) with 95% confidence interval (CI) for A) COVID-19 associated hospitalization. B) Intensive care admission (ICU). C) Progression to lower respiratory tract infection according to SARS-CoV-2 loads categorized in quartiles for comparison. No patient requiring ICU admission had SARS-CoV-2 loads in the lowest quartile.


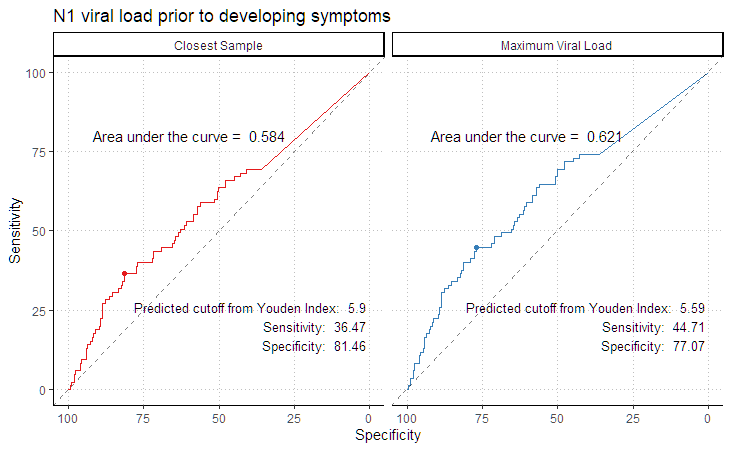

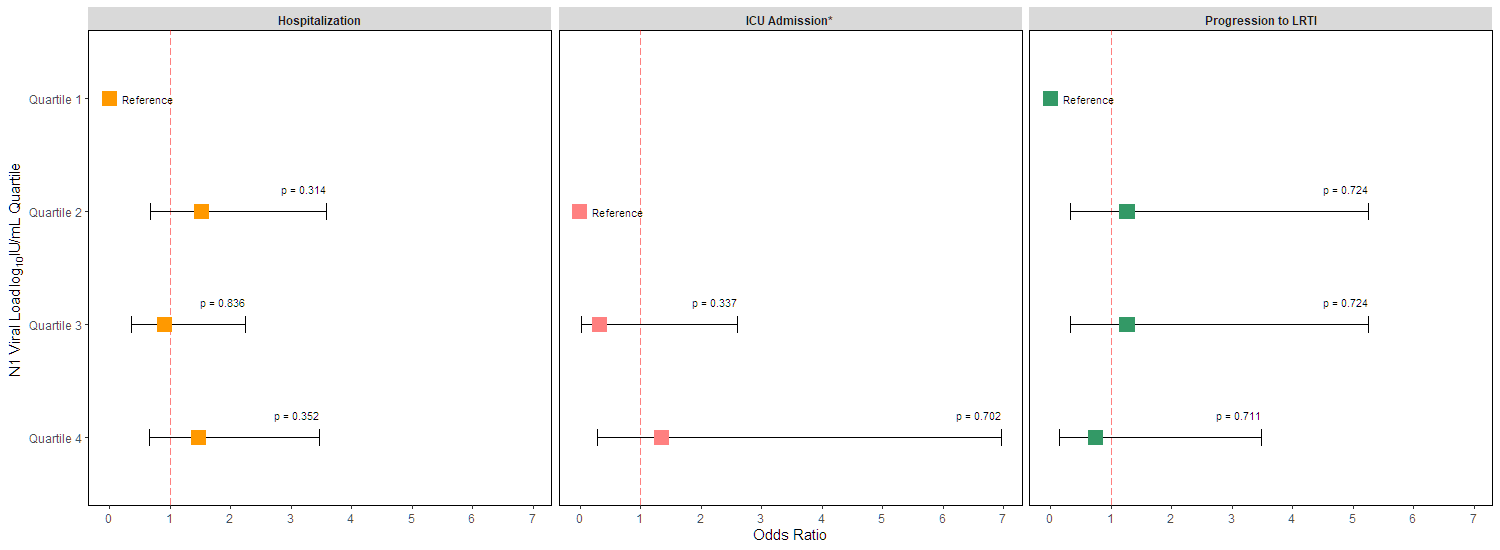

Supplement: piad101_suppl_Supplementary_Figures [file piad101_suppl_supplementary_figures.docx]
