# Supplementary material for: Digital PCR to Measure SARS-CoV-2 RNA, Variants, and Outcomes in Youth
Source: J Pediatric Infect Dis Soc. 2023 Nov 13;12(12):618–26. doi: 10.1093/jpids/piad101 (PMC10725239; doi:10.1093/jpids/piad101)
Supplement: piad101_suppl_Supplementary_Tables [file piad101_suppl_supplementary_tables.docx]

**Digital PCR to Measure SARS-CoV-2 RNA, Variants, and Outcomes in Youth**

Diego R. Hijano, M.D., M.Sc.^1,2^,  Jose A Ferrolino, M.D., M.P.H.^1^, Zhengming Gu, Ph.D.^3^, Jessica N. Brazelton, Ph.D.^3^, Haiqing Zhu, M.D., Ph.D ^3^, Sri Suganda, BS ^3^, Heather L Glasgow, Ph.D.^3^, Ronald H Dallas, Ph.D.^1^ , Kim J Allison, BSN ^1^, Gabriela Maron, M.D., M.S ^1,2^, St Jude COVID-19 Patients Study Team^, Himani Darji ^4^, Li Tang, Ph.D.^4^, Thomas P. Fabrizio, Ph.D. ^1^, Richard J. Webby, Ph.D.^1^, Randall T Hayden M.D.^3^.

^1^ Departments of Infectious Diseases, St. Jude Children’s Research Hospital, Memphis, TN, USA. ^2^ Department of Pediatrics, University of Tennessee Health Sciences Center, Memphis, TN, USA. ^3^Department of Pathology, St. Jude Children’s Research Hospital, Memphis, TN, USA. ^4^Department of Biostatistics, St. Jude Children’s Research Hospital, Memphis, TN, USA.

^St Jude COVID-19 Patients Study Team members: Paige Turner^1^, Megan Peterson^1^, Hailey S. Ross^1^, Madeline Burton^1^, Sapna Pardasani^1^, Jane S Hankins^2, 3^, Clifford Takemoto^2^, Hiroto Inaba^4^, Sara Helmig^4^, Anna Vinitsky^4^, Melissa R Hines^5^, Ali Y Suliman^6^, Paul G Thomas^7^, E Kaitlynn Allen^7^, Joshua Wolf ^1^, Hana Hakim ^1,8,^ Nehali Patel^1^, Katherine Knapp^1^ and Elisabeth E. Adderson^1, 9^.

Departments of ^1^Infectious Diseases, ^2^Hematology, ^3^Global Pediatric Medicine, ^4^Oncology, ^5^Pediatric Medicine, ^6^Bone Marrow Transplant & Cell Therapy, ^7^Immunology St. Jude Children’s Research Hospital, Memphis, TN, USA. ^8^Department of Preventive Medicine, University of Tennessee Health Sciences Center, Memphis, TN, USA. ^9^Department of Pediatrics, University of Tennessee Health Sciences Center, Memphis, TN, USA.

Supplementary Table 1: Symptoms, clinical outcomes, and laboratory values of patients with SARS-CoV-2 over time

|  |  | **0-6 days (n=422)** | **7-13 days (n=67)** | **14-27 days (n=66)** | **> 28 days (n=22)** |
| --- | --- | --- | --- | --- | --- |
| **Variable** |  | **n (%)** | **n (%)** | **n (%)** | **n (%)** |
| Fever | No | 290 (68.72%) | 30 (44.78%) | 28 (42.42%) | 11 (50.00%) |
|  | Yes | 132 (31.28%) | 37 (55.22%) | 38 (57.58%) | 11 (50.00%) |
| Headache | No | 382 (90.52%) | 56 (83.58%) | 56 (84.85%) | 19 (86.36%) |
|  | Yes | 40 (9.48%) | 11 (16.42%) | 10 (15.15%) | 3 (13.64%) |
| Cough | No | 286 (67.77%) | 32 (47.76%) | 35 (53.03%) | 10 (45.45%) |
|  | Yes | 136 (32.23%) | 35 (52.24%) | 31 (46.97%) | 12 (54.55%) |
| Sore Throat | No | 388 (91.94%) | 55 (82.09%) | 61 (92.42%) | 21 (95.45%) |
|  | Yes | 34 (8.06%) | 12 (17.91%) | 5 (7.58%) | 1 (4.55%) |
| Loss of Taste and Smell | No | 401 (95.02%) | 64 (95.52%) | 58 (87.88%) | 20 (90.91%) |
|  | Yes | 21 (4.98%) | 3 (4.48%) | 8 (12.12%) | 2 (9.09%) |
| Shortness of Breath | No | 405 (95.97%) | 61 (91.04%) | 59 (89.39%) | 20 (90.91%) |
|  | Yes | 17 (4.03%) | 6 (8.96%) | 7 (10.61%) | 2 (9.09%) |
| Diarrhea | No | 404 (95.73%) | 62 (92.54%) | 56 (84.85%) | 19 (86.36%) |
|  | Yes | 18 (4.27%) | 5 (7.46%) | 10 (15.15%) | 3 (13.64%) |
| LRTI/URTI | LRTI | 15 (3.55%) | 4 (5.97%) | 5 (7.58%) | 1 (4.55%) |
|  | None | 268 (63.51%) | 39 (58.21%) | 30 (45.45%) | 10 (45.45%) |
|  | URTI | 139 (32.94%) | 24 (35.82%) | 31 (46.97%) | 11 (50.00%) |
| Hospitalization | Already hospitalized | 21 (4.98%) | 8 (11.94%) | 8 (12.12%) | 1 (4.55%) |
|  | No | 360 (85.31%) | 45 (67.16%) | 45 (68.18%) | 18 (81.82%) |
|  | Yes | 41 (9.72%) | 14 (20.90%) | 13 (19.70%) | 3 (13.64%) |
| ICU | No | 55 (13.03%) | 19 (28.36%) | 17 (25.76%) | 1 (4.55%) |
|  | Not hospitalized | 360 (85.31%) | 45 (67.16%) | 45 (68.18%) | 18 (81.82%) |
|  | Yes | 7 (1.66%) | 3 (4.48%) | 4 (6.06%) | 3 (13.64%) |
| Death | No | 420 (99.53%) | 67 (100.0%) | 65 (98.48%) | 22 (100.0%) |
|  | Yes | 2 (0.47%) | 0 | 1 (1.52%) | 0 |
| Treatment Convalescent Plasma | No | 420 (99.53%) | 66 (98.51%) | 64 (96.97%) | 20 (90.91%) |
|  | Yes | 2 (0.47%) | 1 (1.49%) | 2 (3.03%) | 2 (9.09%) |
| Treatment Remdesivir | No | 401 (95.02%) | 61 (91.04%) | 58 (87.88%) | 20 (90.91%) |
|  | Yes | 21 (4.98%) | 6 (8.96%) | 8 (12.12%) | 2 (9.09%) |
| Treatment Steroid | Missing | 10 (2.37%) | 3 (4.48%) | 3 (4.55%) | 0 |
|  | No | 402 (95.26%) | 61 (91.04%) | 57 (86.36%) | 21 (95.45%) |
|  | Yes | 10 (2.37%) | 3 (4.48%) | 6 (9.09%) | 1 (4.55%) |
| Vaccination | No | 329 (77.96%) | 54 (80.60%) | 57 (86.36%) | 21 (95.45%) |
|  | Yes | 93 (22.04%) | 13 (19.40%) | 9 (13.64%) | 1 (4.55%) |
| Sequencing | Alpha | 12 (2.84%) | 0 | 0 | 0 |
|  | Ancestral | 91 (21.56%) | 11 (16.42%) | 15 (22.73%) | 3 (13.64%) |
|  | Delta | 57 (13.51%) | 5 (7.46%) | 2 (3.03%) | 1 (4.55%) |
|  | Epsilon | 1 (0.24%) | 1 (1.49%) | 1 (1.52%) | 0 |
|  | Omicron | 194 (45.97%) | 32 (47.76%) | 20 (30.30%) | 3 (13.64%) |
|  | Undetermined | 65 (15.40%) | 14 (20.90%) | 23 (34.85%) | 12 (54.55%) |
|  | Missing | 2 (0.47%) | 4 (5.97%) | 5 (7.58%) | 3 (13.64%) |
| ALC | Mean (SD) | 2381.00 (2219.15) | 1333.38 (1502.26) | 1899.27 (2092.76) | 2184.58 (2451.60) |
|  | Median (IQR) | 1953.00  (979.00-3190.00) | 691.00  (342.00-1668.00) | 1218.00  (365.00-2754.00) | 1654.00  (250.00-2714.00) |
| Log_10_ IU/ml SARS-CoV-2 N1 | Mean (SD) | 4.67 (1.85) | 4.21 (1.59) | 3.51 (1.37) | 3.10 (0.97) |
|  | Median (IQR) | 4.66 (2.49-6.36) | 3.69 (2.49-5.49) | 3.04 (2.49-3.84) | 2.49 (2.49-3.77) |

Supplementary Table 2: Comparison of symptoms, clinical outcomes, and laboratory values of patients with SARS-CoV-2 with different diagnosis.

|  |  | **Leukemia/**  **Lymphoma** | **ST/NO** | **SCD** | **HIV** | **Other** | **p value** |
| --- | --- | --- | --- | --- | --- | --- | --- |
| **Variable** | **Categories** | **(n=87)** | **(n=145)** | **(n=140)** | **(n=26)** | **(n=64)** |  |
| Fever | No | 60 (68.97) | 111 (76.55) | 63 (45.00) | 23 (88.46) | 50 (78.12) | <0.0001 |
|  | Yes | 27 (31.03) | 34 (23.45) | 77 (55.00) | 3 (11.54) | 14 (21.88) |  |
| Headache | No | 77 (88.51) | 135 (93.10) | 118 (84.29) | 26 (100.00) | 59 (92.19) | 0.042 |
|  | Yes | 10 (11.49) | 10 (6.90) | 22 (15.71) | 0 (0.00) | 5 (7.81) |  |
| Cough | No | 55 (63.22) | 108 (74.48) | 75 (53.57) | 15 (57.69) | 52 (81.25) | 0.0002 |
|  | Yes | 32 (36.78) | 37 (25.52) | 65 (46.43) | 11 (42.31) | 12 (18.75) |  |
| Sore Throat | No | 81 (93.10) | 134 (92.41) | 122 (87.14) | 21 (80.77) | 62 (96.88) | 0.0541 |
|  | Yes | 6 (6.90) | 11 (7.59) | 18 (12.86) | 5 (19.23) | 2 (3.12) |  |
| Loss of Taste and Smell | No | 82 (94.25) | 136 (93.79) | 132 (94.29) | 22 (84.62) | 64 (100.00) | 0.0727 |
|  | Yes | 5 (5.75) | 9 (6.21) | 8 (5.71) | 4 (15.38) | 0 (0.00) |  |
| Shortness of Breath | No | 83 (95.40) | 141 (97.24) | 129 (92.14) | 25 (96.15) | 62 (96.88) | 0.3226 |
|  | Yes | 4 (4.60) | 4 (2.76) | 11 (7.86) | 1 (3.85) | 2 (3.12) |  |
| Diarrhea | No | 80 (91.95) | 139 (95.86) | 136 (97.14) | 26 (100.00) | 61 (95.31) | 0.3037 |
|  | Yes | 7 (8.05) | 6 (4.14) | 4 (2.86) | 0 (0.00) | 3 (4.69) |  |
| LRTI/URTI | LRTI | 3 (3.45) | 1 (0.69) | 11 (7.86) | 0 (0.00) | 2 (3.12) | 0.0114 |
|  | None | 52 (59.77) | 88 (60.69) | 77 (55.00) | 18 (69.23) | 49 (76.56) |  |
|  | URTI | 32 (36.78) | 56 (38.62) | 52 (37.14) | 8 (30.77) | 13 (20.31) |  |
| Hospitalization | Already hospitalized | 5 (5.75) | 19 (13.10) | 2 (1.43) | 0 (0.00) | 0 (0.00) | <0.0001 |
|  | No | 75 (86.21) | 119 (82.07) | 105 (75.00) | 26 (100.00) | 60 (93.75) |  |
|  | Yes | 7 (8.05) | 7 (4.83) | 33 (23.57) | 0 (0.00) | 4 (6.25) |  |
| ICU | No | 10 (11.49) | 25 (17.24) | 31 (22.14) | 0 (0.00) | 3 (4.69) | 0.0104 |
|  | Not hospitalized | 75 (86.21) | 119 (82.07) | 105 (75.00) | 26 (100.00) | 60 (93.75) |  |
|  | Yes | 2 (2.30) | 1 (0.69) | 4 (2.86) | 0 (0.00) | 1 (1.56) |  |
| Death | No | 86 (98.85) | 144 (99.31) | 140 (100.00) | 26 (100.00) | 64 (100.00) | 0.6884 |
|  | Yes | 1 (1.15) | 1 (0.69) | 0 (0.00) | 0 (0.00) | 0 (0.00) |  |
| Treatment convalescent Plasma | No | 85 (97.70) | 145 (100.00) | 140 (100.00) | 26 (100.00) | 64 (100.00) | 0.0702 |
|  | Yes | 2 (2.30) | 0 (0.00) | 0 (0.00) | 0 (0.00) | 0 (0.00) |  |
| Treatment remdesivir | No | 81 (93.10) | 141 (97.24) | 128 (91.43) | 26 (100.00) | 62 (96.88) | 0.1125 |
|  | Yes | 6 (6.90) | 4 (2.76) | 12 (8.57) | 0 (0.00) | 2 (3.12) |  |
| Treatment Steroid | No | 81 (95.29) | 141 (100.00) | 131 (94.93) | 26 (100.00) | 61 (98.39) | 0.0536 |
|  | Yes | 4 (4.71) | 0 (0.00) | 7 (5.07) | 0 (0.00) | 1 (1.61) |  |
| Vaccination | No | 64 (73.56) | 122 (84.14) | 116 (82.86) | 14 (53.85) | 53 (82.81) | 0.0032 |
|  | Yes | 23 (26.44) | 23 (15.86) | 24 (17.14) | 12 (46.15) | 11 (17.19) |  |
| Sequencing | Alpha | 6 (6.90) | 3 (2.07) | 3 (2.14) | 0 (0.00) | 0 (0.00) | 0.233 |
|  | Ancestral | 24 (27.59) | 29 (20.00) | 35 (25.00) | 5 (19.23) | 7 (10.94) |  |
|  | Delta | 11 (12.64) | 21 (14.48) | 13 (9.29) | 4 (15.38) | 10 (15.62) |  |
|  | Epsilon | 0 (0.00) | 1 (0.69) | 0 (0.00) | 0 (0.00) | 0 (0.00) |  |
|  | Omicron | 35 (40.23) | 59 (40.69) | 63 (45.00) | 10 (38.46) | 33 (51.56) |  |
|  | Undetermined | 11 (12.64) | 32 (22.07) | 26 (18.57) | 7 (26.92) | 14 (21.88) |  |
| ALC | Mean (SD) | 1121.89 (1094.80) | 2150.39 (1823.16) | 3234.00 (2724.69) | 2430.83 (1217.10) | 2640.67 (1901.26) | <0.0001 |
|  | Median (IQR) | 732  [355.75, 1486.50] | 1689  [834.50, 3017.50] | 2762.50  [1705.25, 3693.50] | 2157  [1486.50, 3398.25] | 2040  [1388.00, 3596.50] | <0.0001 |
| Log_10_ IU/ml SARS-CoV-2 N1 | Mean (SD) | 4.88 (1.93) | 4.42 (1.81) | 4.41 (1.77) | 4.49 (1.97) | 4.66 (1.92) | 0.3284 |
|  | Median (IQR) | 4.89 [3.08, 6.46] | 4.17 [2.49, 5.80] | 4.06 [2.49, 5.84] | 5.03 [2.49, 5.42] | 4.70 [2.49, 6.38] | 0.35 |

Supplementary Table 3: Symptoms, clinical outcomes, and laboratory values of patients with SARS-CoV-2 over time by variants of concern (VOC).

| **Variables** | | **SARS-CoV-2 variant** | | | | **P value** |
| --- | --- | --- | --- | --- | --- | --- |
|  |  | **Alpha (n=12)** | **Ancestral (n=91)** | **Delta (n=57)** | **Omicron (n=194)** |  |
| **Fever** | No | 11(91.7) | 54(59.3) | 45(78.9) | 120(61.9) | 0.0132 |
|  | Yes | 1(8.33) | 37(40.7) | 12(21.1) | 74(38.1) |  |
| **Headache** | No | 10(83.3) | 81(89.0) | 51(89.5) | 174(89.7) | 0.9219 |
|  | Yes | 2(16.7) | 10(11.0) | 6(10.5) | 20(10.3) |  |
| **Cough** | No | 10(83.3) | 58(63.7) | 38(66.7) | 123(63.4) | 0.5518 |
|  | Yes | 2(16.7) | 33(36.3) | 19(33.3) | 71(36.6) |  |
| **Sore throat** | No | 11(91.7) | 84(92.3) | 56(98.2) | 171(88.1) | 0.1240 |
|  | Yes | 1(8.33) | 7(7.69) | 1(1.75) | 23(11.9) |  |
| **Loss of Taste and Smell** | No | 12(100) | 82(90.1) | 52(91.2) | 188(96.9) | 0.0652 |
|  | Yes |  | 9(9.89) | 5(8.77) | 6(3.09) |  |
| **Shortness of Breath** | No | 12(100) | 86(94.5) | 55(96.5) | 184(94.8) | 0.9686 |
|  | Yes |  | 5(5.49) | 2(3.51) | 10(5.15) |  |
| **Diarrhea** | No | 12(100) | 82(90.1) | 53(93.0) | 191(98.5) | 0.0076 |
|  | Yes |  | 9(9.89) | 4(7.02) | 3(1.55) |  |
| **LRTI/URTI** | LRTI | 1(8.33) | 2(2.20) | 3(5.26) | 8(4.12) | <.0001 |
|  | None | 8(66.7) | 34(37.4) | 41(71.9) | 137(70.6) |  |
|  | URTI | 3(25.0) | 55(60.4) | 13(22.8) | 49(25.3) |  |
| **Hospitalization** | Already hospitalized for reasons |  | 8(8.79) | 1(1.75) | 10(5.15) | 0.6850 |
|  | No | 11(91.7) | 73(80.2) | 51(89.5) | 163(84.0) |  |
|  | Yes | 1(8.33) | 10(11.0) | 5(8.77) | 21(10.8) |  |
| **ICU** | No | 1(8.33) | 15(16.5) | 6(10.5) | 27(13.9) | 0.7951 |
|  | Not hospitalized | 11(91.7) | 73(80.2) | 51(89.5) | 163(84.0) |  |
|  | Yes |  | 3(3.30) |  | 4(2.06) |  |
| **Death** | No | 12(100) | 90(98.9) | 57(100) | 194(100) | 0.4520 |
|  | Yes |  | 1(1.10) |  |  |  |
| **Log_10_ IU/ml SARS-CoV-2 N1** | Mean (SD) | 4.75 (1.98) | 5.31 (1.97) | 4.77 (1.58) | 4.99 (1.69) | 0.2767 |
|  | Median (IQR) | 5.05  [2.49, 6.30] | 5.42  [3.38, 6.93] | 4.78  [3.45, 6.05] | 5.16  [3.31, 6.38] | 0.2779 |

Supplementary Table 4: SARS-CoV-2 loads and vaccination prior to infection.

|  | **Log SARS-CoV-2 N1**  **Median [IQR]** | **P value** |
| --- | --- | --- |
| **Any Vaccination** |  |  |
| No | 4.64 [2.49, 6.41] | 0.6529 |
| Yes | 5.00 [2.49, 6.03] |  |
| **Pfizer-BioNTech** |  |  |
| No | 3.94 [2.49, 5.62] | 0.2153 |
| Yes | 5.20 [2.84, 6.20] |  |
| **Moderna** |  |  |
| No | 5.06 [2.84, 5.99] | 0.4567 |
| Yes | 5.01 [2.90, 5.97] |  |
| **Jansenn** |  |  |
| No | 5.20 [2.49, 6.31] | 0.3077 |
| Yes | 4.29 [2.84, 5.36] |  |
| **Number of Vaccine Doses** |  |  |
| 1 | 4.30 [2.49, 5.61] | 0.1696 |
| 2 | 5.41 [3.12, 6.36] |  |
| 3 | 3.15 [2.49, 5.51] |  |
| 4 | 2.80 [2.80, 2.80] |  |
| **Number of Vaccine Doses** |  |  |
| < 2 | 5.11 [2.84, 6.08] |  |
| >= 2 | 2.97 [2.49, 5.46] |  |

Supplementary Table 5: Time and SARS-CoV-2 VOC in patients with more than one episode.

| **Case** | **SARS-CoV-2 variant for initial infection** | **SARS-CoV-2 variant for reinfection** | **Days from initial infection to reinfection** |
| --- | --- | --- | --- |
| **1** | Ancestral | Omicron | 577 |
| **2** | Undetermined | Ancestral | 104 |
| **3** | Ancestral | Omicron | 551 |
| **4** | Ancestral | Ancestral | 66 |
| **5** | Undetermined | Ancestral | 47 |
| **6** | Ancestral | Omicron | 525 |
| **7** | Undetermined | Omicron | 552 |
| **8** | Ancestral | Omicron | 481 |
| **9** | Ancestral | Undetermined | 483 |
| **10** | Ancestral | Omicron | 392 |
| **11** | Ancestral | Omicron | 392 |
| **12** | Ancestral | Omicron | 385 |
| **13** | Undetermined | Omicron | 403 |
| **14** | Ancestral | Omicron | 363 |
| **15** | Alpha | Omicron | 266 |
| **16** | Ancestral | Omicron | 244 |
| **17** | Delta | Omicron | 193 |
| **18** | Delta | Omicron | 140 |
| **19** | Undetermined | Omicron | 150 |
| **20*** | Delta | Omicron | 133 (35) |
| **21** | Undetermined | Delta | 77 |
| **22** | Delta | Delta | 79 |
| **23** | Delta | Omicron | 103 |
| **24** | Delta | Omicron | 125 |
| **25** | Delta | Omicron | 113 |
| **26** | Delta | Undetermined | 63 |
| **27** | No sample available | Omicron | 30 |

*Case 20 had an initial episode with delta. After 32 days and several negative SARS-COV-2 PCR tests, he tested positive again with same VOC. It was unclear whether this was a new episode given that it was only a month apart and the patient had several negative tests in between. Over ninety days after this last positive SARS-CoV-2 infection, the patient tested positive again for SARS-CoV-2 and the VOC was Omicron.

Supplementary Table 6: SARS-CoV-2 loads and prediction of symptomatic disease.

|  | **Unadjusted** | | **Adjusted for age category** | | **Adjusted for cancer** | | **Adjusted for vaccination** | | **Adjusted for sequence** | |
| --- | --- | --- | --- | --- | --- | --- | --- | --- | --- | --- |
| **Closest sample prior to developing symptoms** | **Odds ratio (95% CI)** | **P value** | **Odds ratio (95% CI)** | **P value** | **Odds ratio (95% CI)** | **P value** | **Odds ratio (95% CI)** | **P value** | **Odds ratio (95% CI)** | **P value** |
| Log_10_ IU/ml SARS-CoV-2 N1 | 1.20  (1.04-1.39) | 0.0124 | 1.20  (1.04-1.39) | 0.0130 | 1.18  (1.02-1.37) | 0.0225 | 1.20  (1.04-1.39) | 0.0124 | 1.19  (1.02-1.40) | 0.0313 |
| **Maximum viral load** | **Odds ratio (95% CI)** | **P value** | **Odds ratio (95% CI)** | **P value** | **Odds ratio (95% CI)** | **P value** | **Odds ratio (95% CI)** | **P value** | **Odds ratio (95% CI)** | **P value** |
| Log_10_ IU/ml SARS-CoV-2 N1 | 1.29  (1.11-1.49) | 0.0006 | 1.29  (1.11-1.49) | 0.0006 | 1.27  (1.09-1.46) | 0.0016 | 1.29  (1.11-1.49) | 0.0006 | 1.30  (1.11-1.53) | 0.0011 |

Supplementary Table 7: SARS-CoV-2 loads and prediction of symptomatic disease.

|  | **OR (95% CI)** | **p-value** |
| --- | --- | --- |
| **Closest sample prior to developing symptoms** | | |
| **Maximum Log_10_ SARS-CoV-2 N1** | 1.18 (1.02 – 1.37) | 0.0255 |
| **Cancer (yes vs no)** | 1.93 (1.10 – 3.39) | 0.0214 |
|  | **OR (95% CI)** | **p-value** |
| **Maximum viral load prior to developing symptoms** | | |
| **Maximum Log_10_ SARS-CoV-2 N1** | 1.27 (1.09 – 1.47) | 0.0017 |
| **Cancer (yes vs no)** | 1.84 (1.05 – 3.25) | 0.0346 |

A multivariate logistic regression model was run adjusted for age, gender, and race. The adjusted variables

with p value <0.1 were selected in the model in a stepwise model selection procedure

Supplementary Table 8: Area under the curve (AUC) and cut point of maximum viral load for asymptomatic patients which became symptomatic versus patients who ed remain asymptomatic throughout infection.

| **Closest sample prior to developing symptoms** | | **AUC (95% CI)** | **Viral load value**  **Cut off (Youden’s J Index)** | **Sensitivity** | **Specificity** |
| --- | --- | --- | --- | --- | --- |
| Asymptomatic turning symptomatic (Yes vs No) | Log_10_ SARS-CoV-2 N1 | 0.58 (0.51 – 0.66) | 5.91 | 36.6% | 82.5% |
| **Maximum viral load prior to developing symptoms** | | **AUC (95% CI)** | **Viral load value**  **Cut off (Youden’s J Index)** | **Sensitivity** | **Specificity** |
| Asymptomatic turning symptomatic (Yes vs No) | Log_10_ SARS-CoV-2 N1 | 0.62 (0.55 – 0.69) | 5.59 | 44.7% | 77.1% |

Supplementary Table 9: Initial and maximum SARS-CoV-2 loads and symptoms.

|  | **Odds ratio**  **(95% CI)** | **P value** | **Odds ratio**  **(95% CI) adjusted for age** | **P value** | **Odds ratio**  **(95% CI) adjusted for race^&^** | **P value** | **Odds ratio**  **(95% CI) adjusted for cancer** | **P value** | **Odds ratio**  **(95% CI) adjusted for chemotherapy** | **P values** | **Odds ratio**  **(95% CI) adjusted for Lymphocyte counts (log value)** | **P values** | **Odds ratio**  **(95% CI) adjusted for sequencing variant^** | **P values** | **Odds ratio (95% CI) adjusted for vaccine status** | **P value** |
| --- | --- | --- | --- | --- | --- | --- | --- | --- | --- | --- | --- | --- | --- | --- | --- | --- |
| **Initial SARS-CoV-2 N1** | | | | | | | | | | | | | | | | |
| Any symptoms | 1.34  (1.20-1.50) | <0.0001 | 1.36  (1.21-1.52) | <0.0001 | 1.35  (1.20-1.51) | <0.0001 | 1.34  (1.20-1.50) | <0.0001 | 1.32  (1.18-1.48) | <0.0001 | 1.33  (1.17-1.50) | <0.0001 | 1.26  (1.12-1.42) | 0.0002 | 1.34  (1.20-1.50) | <0.0001 |
| Fever | 1.35  (1.21-1.52) | <0.0001 | 1.34  (1.19-1.51) | <0.0001 | 1.37 (1.21-1.54) | <0.0001 | 1.37  (1.21-1.54) | <0.0001 | 1.36 (1.21-1.53) | <0.0001 | 1.35  (1.19-1.53) | <0.0001 | 1.24  (1.10-1.41) | 0.0008 | 1.35  (1.20-1.52) | <0.0001 |
| Cough | 1.25  (1.11-1.40) | 0.0001 | 1.25  (1.12-1.41) | 0.0001 | 1.25 (1.12-1.41) | 0.0001 | 1.25  (1.12-1.40) | 0.0001 | 1.24 (1.11-1.39) | 0.0003 | 1.22  (1.08-1.38) | 0.0016 | 1.18  (1.04-1.33) | 0.0106 | 1.25  (1.12-1.41) | 0.0001 |
| Shortness of Breath | 1.00  (0.77-1.31) | 0.9717 | 1.00  (0.77-1.31) | 0.9906 | 1.00 (0.76-1.31) | 0.9796 | 1.01  (0.77-1.31) | 0.9553 | 1.00 (0.77-1.31) | 0.9859 | 0.99  (0.76-1.30) | 0.9638 | 0.88  (0.66-1.16) | 0.3643 | 1.01  (0.77-1.31) | 0.9598 |
| **Maximum SARS-CoV-2 N1** | | | | | | | | | | | | | | | | |
| Any symptoms | 1.30  (1.17-1.44) | <0.0001 | 1.31  (1.17-1.46) | <0.0001 | 1.31  (1.18-1.46) | <0.0001 | 1.31  (1.17-1.45) | <0.0001 | 1.28  (1.15-1.42) | <0.0001 | 1.30  (1.16-1.47) | <0.0001 | 1.25  (1.11-1.41) | 0.0002 | 1.30  (1.17-1.44) | <0.0001 |
| Fever | 1.28  (1.15-1.43) | <0.0001 | 1.27  (1.14-1.42) | <0.0001 | 1.30  (1.17-1.46) | <0.0001 | 1.30  (1.17-1.46) | <0.0001 | 1.29  (1.15-1.44) | <0.0001 | 1.29  (1.15-1.45) | <0.0001 | 1.23  (1.09-1.39) | 0.0007 | 1.29  (1.16-1.44) | <0.0001 |
| Cough | 1.20  (1.07-1.33) | 0.0011 | 1.20  (1.07-1.33) | 0.0011 | 1.21  (1.08-1.34) | 0.0008 | 1.21  (1.08-1.34) | 0.0007 | 1.19  (1.07-1.33) | 0.0016 | 1.18  (1.05-1.32) | 0.0065 | 1.15  (1.02-1.30) | 0.0196 | 1.20  (1.07-1.33) | 0.0011 |
| Shortness of Breath | 0.90  (0.71-1.16) | 0.4179 | 0.89  (0.69-1.14) | 0.3592 | 0.91  (0.70-1.16) | 0.4348 | 0.91  (0.71-1.17) | 0.4717 | 0.90  (0.70-1.15) | 0.3934 | 0.91  (0.70-1.18) | 0.4713 | 0.83  (0.64-1.09) | 0.1859 | 0.90  (0.71-1.15) | 0.4146 |

^&^ Race here is White and Asian vs Black; ^ Sequencing variant is grouped as Omicron, Ancestral, Delta and Others (Alpha, Epsilon and Undetermined)

Supplementary Table 10: Multivariate model of initial SARS-CoV-2 loads predicting clinical outcomes upon initial presentation.

|  | **SARS-CoV-2 N1** | |
| --- | --- | --- |
|  | **Odds ratio (95% CI)** | **P value** |
| **Any Symptom** |  |  |
| **Viral Load** | 1.32 (1.16-1.49) | <0.0001 |
| **Lymphocyte count** | 0.49 (0.28-0.85) | 0.0105 |
| **Race** |  |  |
| **Race (Black vs White)** | 2.41 (1.47-3.95) | 0.0005 |
| **Race (Multiracial vs White)** | 1.66 (0.61-4.51) | 0.3227 |
| **Fever** |  |  |
| **Viral Load** | 1.26 (1.10-1.45) | 0.0010 |
| **Race (Black vs White)** | 2.10 (1.26-3.49) | 0.0046 |
| **Race (Multiracial vs White)** | 1.77 (0.58-5.44) | 0.3509 |
| **Sequencing Variant (Ancestral vs Others)** | 2.83 (1.10-7.27) | 0.0122 |
| **Sequencing Variant (Delta vs Others)** | 1.48 (0.50-4.36) | 0.4387 |
| **Sequencing Variant (Omicron vs Others)** | 4.10 (1.72-9.79) | 0.0050 |
| **Vaccination (Yes vs No)** | 0.35 (0.18-0.68) | 0.0018 |
| **Cough** |  |  |
| **Viral Load** | 1.22 (1.08-1.38) | 0.0019 |
| **Race (Black vs White)** | 2.40 (1.42-4.05) | 0.0011 |
| **Race (Multiracial vs White)** | 1.98 (0.71-5.55) | 0.1916 |
| **Chemotherapy (Yes vs No)** | 1.69 (0.96-2.96) | 0.0668 |
| **Shortness of Breath** |  |  |
| **Viral Load** | 0.99 (0.75-1.29) | 0.9322 |
| **Respiratory Tract Infection** |  |  |
| **Viral Load** | 1.23 (1.07-1.41) | 0.0027 |
| **Sequencing Variant (Ancestral vs Others)** | 3.06 (1.40-6.65) | 0.0049 |
| **Sequencing Variant (Delta vs Others)** | 0.74 (0.30-1.78) | 0.4966 |
| **Sequencing Variant (Omicron vs Others)** | 0.72 (0.36-1.43) | 0.3448 |
| **Hospitalization (Non COVID-19 vs hospitalization vs not hospitalized)** |  |  |
| **Viral Load** | 1.01 (0.78-1.30) | 0.9529 |
| **Race (Black vs White)** | 0.29 (0.08-1.11) | 0.0710 |
| **Race (Multiracial vs White)** | 2.21 (0.53-9.18) | 0.2767 |
| **Chemotherapy (Yes vs No)** | 4.14 (1.50-11.46) | 0.0062 |
| **Hospitalization (COVID-19 hospitalization vs not hospitalized)** |  |  |
| **Viral Load** | 1.10 (0.92-1.33) | 0.2952 |
| **Race (Black vs White)** | 3.84 (1.58-9.32) | 0.0030 |
| **Race (Multiracial vs White)** | 1.22 (0.14-10.71) | 0.8548 |
| **Chemotherapy (Yes vs No)** | 1.21 (0.50-2.91) | 0.6778 |
| **ICU Admission (Yes vs not hospitalized)** |  |  |
| **Viral Load** | 1.45 (0.95-2.21) | 0.0832 |
| **Chemotherapy (Yes vs No)** | 0.47 (0.06-4.04) | 0.4940 |
| **ICU Admission (No vs not hospitalized)** |  |  |
| **Viral Load** | 1.03 (0.87-1.21) | 0.7617 |
| **Chemotherapy (Yes vs No)** | 1.94 (1.03-3.66) | 0.0413 |

A multivariate stepwise logistic regression model was ran adjusted for age, gender, race, cancer, chemotherapy,

sequencing variant, lymphocyte count and vaccination status. The adjusted variables with p value <0.1 were selected in the model.

Supplementary Table 11: Multivariate model of maximum SARS-CoV-2 loads predicting clinical outcomes upon initial presentation.

|  | **SARS-CoV-2 N1** | |
| --- | --- | --- |
|  | **Odds ratio (95% CI)** | **P value** |
| **Any Symptom** |  |  |
| **Viral Load** | 1.30 (1.15-1.47) | <0.0001 |
| **Lymphocyte counts** | 0.59 (0.36-0.95) | 0.0285 |
| **Race** |  |  |
| **Black vs White** | 2.26 (1.42-3.62) | 0.0006 |
| **Multiracial vs White** | 1.27 (0.49-3.29) | 0.6268 |
| **Fever** |  |  |
| **Viral Load** | 1.27 (1.11-1.45) | 0.0005 |
| **Race** |  |  |
| **Black vs White** | 2.18 (1.34-3.53) | 0.0016 |
| **Multiracial vs White** | 1.88 (0.69-5.11) | 0.2154 |
| **Sequencing Variant (Ancestral vs Others)** | 1.54 (0.71-3.34) | 0.2774 |
| **Sequencing Variant (Delta vs Others)** | 0.72 (0.28-1.84) | 0.4943 |
| **Sequencing Variant (Omicron vs Others)** | 1.88 (0.93-3.82) | 0.0809 |
| **Vaccination (Yes vs No)** | 0.34 (0.18-0.64) | 0.0009 |
| **Cough** |  |  |
| **Viral Load** | 1.19 (1.05-1.33) | 0.0045 |
| **Race** |  |  |
| **Black vs White** | 2.11 (1.32-3.36) | 0.0017 |
| **Multiracial vs White** | 1.11 (0.40-3.07) | 0.8432 |
| **Shortness of Breath** |  |  |
| **Viral Load** | 0.92 (0.71-1.19) | 0.5309 |
| **Cancer (Yes vs No)** | 0.38 (0.14-1.03) | 0.0583 |
| **Respiratory Tract Infection** |  |  |
| **Viral Load** | 1.20 (1.05-1.37) | 0.0059 |
| **Sequencing Variant (Ancestral vs Others)** | 2.06 (1.01-4.17) | 0.0457 |
| **Sequencing Variant (Delta vs Others)** | 0.48 (0.21-1.11) | 0.0856 |
| **Sequencing Variant (Omicron vs Others)** | 0.53 (0.28-1.00) | 0.0495 |
| **Hospitalization (Non COVID-19 vs hospitalization vs not hospitalized)** |  |  |
| **Viral Load** | 0.96 (0.76-1.21) | 0.7126 |
| **Cancer (Yes vs No)** | 18.15 (2.41-136.9) | 0.0049 |
| **Hospitalization (COVID-19 hospitalization vs not hospitalized)** |  |  |
| **Viral Load** | 1.08 (0.91-1.28) | 0.3918 |
| **Cancer (Yes vs No)** | 0.42 (0.21-0.81) | 0.0096 |
| **ICU Admission (Yes vs Not Hosp)** |  |  |
| **Viral Load** | 1.02 (0.88-1.18) | 0.8406 |
| **ICU Admission (No vs Not Hosp)** |  |  |
| **Viral Load** | 1.21 (0.81-1.81) | 0.3403 |

A multivariate stepwise logistic regression model was ran adjusted for age, gender, race, cancer, chemotherapy,

sequencing variant, lymphocyte count and vaccination status. The adjusted variables with p value <0.1 were selected in the model.

Supplementary Table 12: Maximum SARS-CoV-2 loads and clinical outcomes.

|  | **Odds ratio**  **(95% CI)** | **P value** | **Odds ratio**  **(95% CI) adjusted for age** | **P value** | **Odds ratio**  **(95% CI) adjusted for race** | **P value** | **Odds ratio**  **(95% CI) adjusted for cancer** | **P value** | **Odds ratio**  **(95% CI) adjusted for chemotherapy** | **P value** | **Odds ratio**  **(95% CI) adjusted for Lymphocyte counts (log value)** | **P value** | **Odds ratio**  **(95% CI) adjusted for sequencing variant** | **P value** | **Odds ratio**  **(95% CI) adjusted for vaccine status** | **P value** |
| --- | --- | --- | --- | --- | --- | --- | --- | --- | --- | --- | --- | --- | --- | --- | --- | --- |
| **Maximum SARS-CoV-2 N1** | | | | | | | | | | | | | | | | |
| URTI/LRTI |  |  |  |  |  |  |  |  |  |  |  |  |  |  |  |  |
| RTI vs None | 1.16  (1.04-1.28) | 0.0062 | 1.15  (1.04-1.28) | 0.0077 | 1.16  (1.05-1.29) | 0.0050 | 1.15  (1.04-1.28) | 0.0071 | 1.15  (1.04-1.28) | 0.0085 | 1.16  (1.04-1.30) | 0.0090 | 1.20  (1.06-1.36) | 0.0033 | 1.16  (1.04-1.28) | 0.0059 |
| Hospitalization |  |  |  |  |  |  |  |  |  |  |  |  |  |  |  |  |
| Yes vs No | 1.03 (0.88-1.21) | 0.7050 | 1.03  (0.87-1.21) | 0.7598 | 1.04  (0.88-1.23) | 0.6436 | 1.05  (0.89-1.23) | 0.5876 | 1.03  (0.87-1.21) | 0.7609 | 1.04  (0.88-1.23) | 0.6307 | 1.02  (0.85-1.23) | 0.8114 | 1.03  (0.88-1.21) | 0.7078 |
| ICU admission |  |  |  |  |  |  |  |  |  |  |  |  |  |  |  |  |
| Not admitted to ICU vs Not Hospitalized | 1.02  (0.88-1.18) | 0.7965 | 1.00  (0.86-1.16) | 0.9972 | 1.02  (0.89-1.18) | 0.7636 | 1.02  (0.88-1.17) | 0.8346 | 0.98  (0.84-1.13) | 0.7584 | 1.00  (0.86-1.16) | 0.9861 | 0.99  (0.84-1.16) | 0.9068 | 1.02  (0.88-1.18) | 0.7940 |
| Yes vs Not hospitalized | 1.24  (0.83-1.85) | 0.3003 | 1.23  (0.82-1.85) | 0.3064 | 1.24  (0.83-1.87) | 0.2942 | 1.26  (0.84-1.90) | 0.2566 | 1.30  (0.87-1.94) | 0.2020 | 1.22  (0.82-1.83) | 0.3327 | 1.07  (0.71-1.63) | 0.7372 | 1.24  (0.83-1.85) | 0.3008 |

RTI: respiratory tract infection; ^&^ Race here is White and Asian vs Black; ^ Sequencing variant is grouped as Omicron, Ancestral, Delta and Others (Alpha, Epsilon and Undetermined)

## Supplementary Table 13: Mixed effect models for SARS-CoV-2 load predicting symptoms and clinical outcomes.

|  | **Odds ratio**  **(95% CI)** | **P value** | **Odds ratio**  **(95% CI) adjusted for age** | **P value** | **Odds ratio**  **(95% CI) adjusted for race^&^** | **P value** | **Odds ratio**  **(95% CI) adjusted for cancer** | **P value** | **Odds ratio**  **(95% CI) adjusted for chemotherapy** | **P values** | **Odds ratio**  **(95% CI) adjusted for Lymphocyte counts (log value)** | **P values** | **Odds ratio**  **(95% CI) adjusted for sequencing variant^** | **P values** | **Odds ratio (95% CI) adjusted for vaccine status** | **P value** |
| --- | --- | --- | --- | --- | --- | --- | --- | --- | --- | --- | --- | --- | --- | --- | --- | --- |
| **SARS-CoV-2 N1** | | | | | | | | | | | | | | | | |
| Any symptoms | 1.17  (1.03 - 1.33) | 0.0195 | 1.17  (1.03 - 1.33) | 0.0190 | 1.18  (1.04 - 1.35) | 0.0128 | 1.17  (1.03 - 1.33) | 0.0196 | 1.15  (1.01 - 1.31) | 0.0345 | 1.17  (1.02 - 1.35) | 0.0277 | 1.19  (1.04 - 1.36) | 0.0103 | 1.17  (1.02 - 1.33) | 0.0201 |
| Fever | 1.17  (1.03 - 1.34) | 0.0189 | 1.17  (1.02 - 1.33) | 0.0256 | 1.19  (1.04 - 1.37) | 0.0128 | 1.18  (1.03 - 1.35) | 0.0181 | 1.18  (1.03 - 1.35) | 0.0172 | 1.19  (1.03 - 1.37) | 0.0184 | 1.2  (1.05 - 1.38) | 0.0078 | 1.17  (1.02 - 1.34) | 0.0224 |
| Cough | 1.08  (0.95 - 1.24) | 0.2299 | 1.09  (0.95 - 1.24) | 0.2224 | 1.1  (0.96 - 1.26) | 0.1775 | 1.09  (0.95 - 1.24) | 0.2271 | 1.08  (0.95 - 1.24) | 0.2389 | 1.07  (0.93 - 1.23) | 0.3500 | 1.09  (0.95 - 1.24) | 0.2278 | 1.09  (0.95 - 1.24) | 0.2174 |
| Shortness of Breath | 0.86  (0.65 - 1.13) | 0.2813 | 0.86  (0.65 - 1.13) | 0.2763 | 0.87  (0.65 - 1.15) | 0.3244 | 0.86  (0.65 - 1.14) | 0.2997 | 0.86  (0.65 - 1.14) | 0.3079 | 0.87  (0.65 - 1.16) | 0.3329 | Did not converge |  | 0.86  (0.65 - 1.14) | 0.2847 |
| RTI vs None | 1.15  (1.01 - 1.31) | 0.0336 | 1.15  (1.01 - 1.32) | 0.0339 | 1.17  (1.02 - 1.33) | 0.0240 | 1.15  (1.01 - 1.31) | 0.0338 | 1.16  (1.01 - 1.32) | 0.0295 | 1.15  (1 - 1.32) | 0.0582 | 1.18  (1.03 - 1.36) | 0.0182 | 1.15  (1.01 - 1.31) | 0.0365 |
| Hospitalization | 0.94  (0.8 - 1.11) | 0.4630 | 0.93  (0.79 - 1.1) | 0.4121 | 0.95  (0.81 - 1.12) | 0.5245 | 0.94  (0.8 - 1.11) | 0.4656 | 0.93  (0.79 - 1.1) | 0.4064 | 0.93  (0.79 - 1.1) | 0.4035 | 0.95  (0.81 - 1.12) | 0.5617 | 0.94  (0.8 - 1.11) | 0.4609 |
| ICU admission | 1.08  (0.91 - 1.27) | 0.4005 | 1.09  (0.92 - 1.29) | 0.3335 | 1.07  (0.9 - 1.27) | 0.4699 | 1.07  (0.91 - 1.27) | 0.4022 | 1.09  (0.92 - 1.29) | 0.3389 | 1.09  (0.91 - 1.3) | 0.3341 | 1.07  (0.9 - 1.27) | 0.4433 | 1.08  (0.91 - 1.28) | 0.3959 |

RTI: respiratory tract infection; ^&^ Race here is White and Asian vs Black; ^ Sequencing variant is grouped as Omicron, Ancestral, Delta and Others (Alpha, Epsilon and Undetermined). Covariance matrix structure used in the model is Compound Symmetry or Auto Regressive

Supplementary Table 14: Multivariate mixed effect models of SARS-CoV-2 load predicting clinical outcomes.

|  | **SARS-CoV-2 N1** | |
| --- | --- | --- |
|  | **Odds ratio (95% CI)** | **P value** |
| **Any Symptom** |  |  |
| **Viral Load** | 1.19 (1.03 – 1.34) | 0.0233 |
| **Lymphocyte count** | 0.55 (0.34 – 0.88) | 0.0136 |
| **Race (Black vs White)** | 1.79 (1.03 – 3.15) | 0.0404 |
| **Race (Multiracial vs White)** | 2.11 (0.57 – 7.77) | 0.2599 |
| **Fever** |  |  |
| **Viral Load** | 1.21 (1.05 – 1.39) | 0.0096 |
| **Cancer (Yes vs No)** | 0.52 (0.31 – 0.87) | 0.0136 |
| **Sequencing Variant (Alpha vs Ancestral)** | 0.12 (0.01 – 1.30) | 0.0816 |
| **Sequencing Variant (Delta vs Ancestral)** | 0.42 (0.18 – 0.99) | 0.0474 |
| **Sequencing Variant (Omicron vs Ancestral)** | 1.26 (0.68 – 2.34) | 0.4552 |
| **Vaccination (Yes vs No)** | 0.32 (0.16 – 0.65) | 0.0016 |
| **Cough** |  |  |
| **Viral Load** | 1.09 (0.96 – 1.26) | 0.1775 |
| **Race (Black vs White)** | 2.02 (1.20 – 3.39) | 0.0080 |
| **Race (Multiracial vs White)** | 1.74 (0.53 – 5.68) | 0.3587 |
| **Shortness of Breath** |  |  |
| **Viral Load** | 0.86 (0.65 – 1.13) | 0.2813 |
| **Respiratory Tract Infection** |  |  |
| **Viral Load** | 1.18 (1.03 – 1.36) | 0.0182 |
| **Sequencing Variant (Ancestral vs Others)** | 0.25 (0.05- 1.17) | 0.0778 |
| **Sequencing Variant (Delta vs Others)** | 0.22 (0.09 – 0.48) | 0.0002 |
| **Sequencing Variant (Omicron vs Others)** | 0.24 (0.13 – 0.42) | <0.0001 |
| **Hospitalization (Yes vs No)** |  |  |
| **Viral Load** | 0.94 (0.80 – 1.11) | 0.4656 |
| **Cancer (Yes vs No)** | 0.92 (0.51 – 1.67) | 0.7828 |
| **ICU Admission (Yes vs No)** |  |  |
| **Viral Load** | 1.08 (0.91 – 1.27) | 0.4005 |

A multivariate stepwise logistic regression model was ran adjusted for age, gender, race, cancer, chemotherapy,

sequencing variant, lymphocyte count and vaccination status. The adjusted variables with p value <0.1 were selected in the model.

Supplementary Table 15: Linear mixed effect models of SARS-CoV-2 loads, symptoms, time interval and interaction terms between time and symptoms.

|  | **SARS-CoV-2 loads, symptoms and time interval** | | **SARS-CoV-2 loads, symptoms, time interval and interaction terms** | |
| --- | --- | --- | --- | --- |
|  | **β - coefficient** | **P value** | **β - coefficient** | **P value** |
| **Any Symptom** | 0.67 (0.35 – 0.98) | <0.0001 | -0.25 ([-1.96] – 1.46) | 0.7732 |
| **Fever** | 0.69 (0.36 – 1.03) | <0.0001 | -0.06 ([-1.64] – 1.52) | 0.9429 |
| **Cough** | 0.49 (0.16 – 0.84) | 0.0040 | 0.19 ([-1.44] – 1.83) | 0.8172 |

Supplementary Table 16: Additional diagnosis for category “Other” in Table 1.

| **Other diagnosis** | **n** | **Percent of Other (n=64)** | **Percent of all subjects (n=462)** |
| --- | --- | --- | --- |
| Benign Tumor | 5 | 7.81% | 1.08% |
| Healthy | 3 | 4.69% | 0.65% |
| Hematologic | 39 | 60.94% | 8.44% |
| Histiocytosis | 5 | 7.81% | 1.08% |
| MDS | 1 | 1.56% | 0.22% |
| Perinatal HIV Exposure | 6 | 9.38% | 1.30% |
| Other | 5 | 7.81% | 1.08% |
